# Electrospun Polymeric Composite Fibers Containing Te-Doped Bioactive Glass Powders

**DOI:** 10.3390/polym17152057

**Published:** 2025-07-28

**Authors:** Marta Miola, Elisa Piatti, Francesco Iorio, Aldo R. Boccaccini, Enrica Verné

**Affiliations:** 1 Department of Applied Science and Technology, Politecnico di Torino, 10129 Turin, Italy; marta.miola@polito.it (M.M.); elisa.piatti@polito.it (E.P.); 2 Institute of Biomaterials, University of Erlangen-Nuremberg, 91058 Erlangen, Germany; francesco.iorio@fau.de (F.I.); aldo.boccaccini@fau.de (A.R.B.)

**Keywords:** poly (ϵ-caprolactone), electrospinning, bioactive glasses, tellurium, antibacterial

## Abstract

In this work, the electrospinning technique was used to prepare novel polymeric composite fibers containing Te-doped bioactive glass powders. Bioactive glass powders containing tellurium (STe5 glass) were chosen as fillers for the composites, owing to their bioactive, antibacterial, and antioxidant properties. The biopolymer poly (ϵ-caprolactone) (PCL) and acetic acid (AA) were used as raw materials for the preparation of the polymeric matrix. FESEM analysis confirmed a good incorporation of the glass powders in the polymeric fibers, of up to 20% by weight. Wettability, mechanical, in vitro stability and preliminary antibacterial tests were also performed. The results showed that the treatment in AA did not affect the bioactivity of the glass powders, the presence of STe5 powders in PCL enhanced the wettability of the fibers, and mechanical properties improved by increasing the amount of STe5 powders, as well as the antibacterial effect. Therefore, the obtained materials appear promising for developing multifunctional composite materials for applications in tissue engineering.

## 1. Introduction

Bioactive glasses (BGs) are a particular class of glasses characterized by so-called bioactivity, which is the well-known ability to form a strong bond with human tissues through the formation of an interface mineral layer of hydroxyapatite (HAp), the mineral phase present in bone [1]. In addition, BGs can be enriched with several ions to overexpress different properties such as osteogenic, pro-angiogenic, antibacterial, and antioxidant, like in the case of strontium [2], boron [3], copper [4], and cerium [5,6], respectively (just to cite a few examples). The interested reader can deepen their knowledge on the effect of doping ions in the numerous reviews available on this interesting topic [7,8,9]. BG particles can be used as fillers in composite materials, such as composite scaffolds like electrospun polymeric fibrous mats, in order to impart the unique properties of BGs to the final composites [10]. The development of new composite multifunctional fibrous materials is of great interest because of the possibility of structurally mimicking the extracellular matrix (ECM) of cells of human tissues [11] and simultaneously performing different functionalities, such as stimulating tissue regeneration and fighting bacterial infections, through antimicrobial and anti-inflammatory effects. On this note, poly (ϵ-caprolactone) (PCL) is a synthetic polyester commonly employed for the fabrication of composite scaffold matrices owing to its processability through the electrospinning technique in benign solvents [12] and its long-term stability in vivo, which led to its FDA approval for various tissue engineering applications [13]. While PCL possesses drawbacks limiting its use as a standalone component for ECM-mimicking scaffolds, primarily its hydrophobicity and lack of bioactivity for specific bone tissue engineering applications [14], it also shows great potential for blends with other polymers or functionalizations with fillers [15,16], ultimately constituting composite scaffolds with enhanced biocompatibility and even additional desirable properties to answer a wide range of biomedical needs. In particular, there is a growing interest in multifunctional biomaterials, possessing both therapeutic and antibacterial/antimicrobial properties, due to the increasing number of micro-organisms resistant to antibiotics [17].

In this work, novel polymeric composite fibers containing a Te-doped BG were produced by the electrospinning technique using benign solvents, with the aim of developing bioactive and antibacterial biomaterials for tissue engineering purposes. In detail, BG powders containing 5 mol% of TeO_2_ (STe5) were selected as fillers, because of its bioactive, antibacterial, and antioxidant properties, already evidenced in previous works [18]. On the basis of the authors’ knowledge, no prior studies have been conducted on composite polymeric fibers containing Te-doped BG particles. In addition, the study of composite materials containing Te ions is still in its early stages, and only a couple of papers have been published so far [19,20], in which poly (methyl methacrylate) was directly loaded with tellurium, and ultrafine fibers were fabricated using pressurized gyration and vitrimeric and biobased scaffolds containing Te-doped glass were developed. Moreover, the experimental work here reported aligns with the framework of “green electrospinning”, which involves applying sustainability principles to biomaterial production through the use of biologically benign solvents, as classified by the U.S. Food and Drug Administration Q3C Class 3.

## 2. Materials and Methods

### 2.1. Materials

Composite fibers containing STe5 BG powders were successfully electrospun using poly (ϵ-caprolactone) (PCL, 80 kDa, Sigma Aldrich, Munich, Germany), acetic acid (AA) at 98% (AA, VWR, Germany), and STe5 glass powders with composition (mol%) 43.6 SiO_2_, 16.7 Na_2_O, 34.2 CaO, 0.5 P_2_O_5_, 5 TeO_2_ [17]. Undoped glass powders (STe0 with composition in mol% 48.6 SiO_2_, 16.7 Na_2_O, 34.2 CaO, 0.5 P_2_O_5_ [18]) were used as control glass, for the evaluation of the effect of Te ions on the spinnability of the electrospinning solution and final properties of the composite fibers. In order to synthesize STe0 and STe5 glasses, the following reagents (purchased from Sigma-Aldrich, Merck, Darmstadt, Germany) were used: SiO_2_, Na_2_CO_3_, CaCO_3_, Ca_3_(PO_4_)_2_ and TeO_2_.

### 2.2. Glass Synthesis

STe0 and STe5 glasses were produced in the form of frits by the traditional melting and quenching technique in distilled water, following a previous protocol of Miola et al. [18]. The melting of the glass reagents was performed in a Pt crucible at 1450 °C for 1 h. In order to obtain glass powders, these frits were ground in a zirconia 6-ball planetary mill (Fritsh, Pulverisette 6, Idar-Oberstein, Germany) for 3 h, inverting the direction of the rotation of the balls every 30 min. Then, glass powders were sieved at 20 µm with a mesh to favor the incorporation of the BG powders into the fibers.

### 2.3. Fiber Synthesis

The final outcome of an electrospinning procedure is strongly influenced by several solution processes, and environmental parameters [21]. Thus, because of the novelty of these Te-doped BGs and this experimental work (on the basis of the authors’ knowledge) being the first investigation of the interaction of Te-containing BG powders with a PCL-based electrospinning solution, process parameters needed to be adapted in order to successfully electrospin composite fibers incorporating Te-doped BGs. Therefore, the effect of different BG amounts, different applied voltage, different working distance, and different flow rates were investigated. The added BG content was varied in the range 5–30 wt%, with respect to PCL content. The limits of this range were selected on the basis of previous experimental studies showing that 5 wt% of BGs (with respect to the PCL amount in the system PCL/AA) resulted in higher in vitro cell viability and bone formation if compared to neat PCL [22], whereas, in the case of 30 wt%, literature results are more trivial, since some studies show that only a percentage of 30 wt% of BGs (with respect to the polymer amount) can successfully impart bioactive properties to the composite electrospun fibers [23,24] and other studies indicate this amount was excessive because the glass fillers were not well dispersed and not stabilized in the PCL/AA solution, agglomerating and affecting both the viscosity and conductivity of the electrospinning solution and the adhesion between BG particles and PCL at interfaces [25,26].

For the fabrication of these composite fibers, BG powders were added to a solution of PCL (at 20 *w*/*v*%) in AA. This PCL solution was prepared, magnetically stirring overnight PCL grains in AA, until they dissolved and a clear solution was obtained, in agreement with previous work by Liverani et al. [25]. Then, to ensure homogeneity, before using it, this PCL solution was ultrasonicated for 1 h in an US bath. Later, when the PCL solution was homogenous, BG powders were poured into the polymeric solution and mixed under ultrasonication for 10 min and then manually for 1 min.

All fibrous samples were electrospun using the horizontal electrospinning setup Starter Kit 40 KV Web (Linari Engineering srl, Valpiana (GR), Italy), which was composed of a syringe pump (BSP-99M Razel), a BD plastic syringe of 3 mL, a flat plastic collector plate, a high voltage generator, and two electrodes, a positive one clamped directly to the needle of the syringe and a negative one attached on the aluminum foil covering the collector to make it conductive. The working distance was defined by the distance between the positive electrode on the needle and the negative electrode on the collector plate. When the electrospinning solution was ready, the syringe was filled with it and placed on the pump, which allowed delivery of the electrospinning solution and control of the mass flow rate during the electrospinning process. The fabrication of the samples was carried out at room temperature (varying in the range 25–28 °C) in air with humidity values in the range 30–50%.

On the basis of the spinnability of the electrospinning solution, fiber deposition rate, thickness of the fibers (for the same spinning time), and presence of the typical roughness given by the incorporation of glass powders in the polymeric mats, the most promising electrospun composite fibrous mats were selected and morphologically analyzed. On the basis of morphological results, a further selection was carried out: composites containing 30 wt% of glass powders were discarded and the best electrospinning parameters for the synthesis of the fibers containing 5 wt%, 10 wt% and 20 wt% of glass powders were chosen. In order to investigate both the influence of glass addition and the effect of the dopant Te ions on the electrospinnability and conductivity of the solution, and on the final properties of the fibers, neat PCL fibers and composite fibers containing STe0 powders were also synthetized. PCL fibers were fabricated following a protocol described in previous works [25,26]. In order to easily compare composite fibrous electrospun membranes with the doped and undoped BGs, STe0-containing fibers were synthetized using the same parameters selected for STe5-containing fibers.

### 2.4. Morphological Characterization of the BG Powders

STe0 and STe5 powders were morphologically characterized using the field-emission scanning electron microscope FESEM Gemini SUPRATM 40 (Zeiss, Oberkochen, Germany), aiming to assess the influence of filler morphology on its incorporation in the polymeric matrix. The samples were prepared by attaching glass powders to a stub by means of double-sided carbon tape and sputtered with a layer of platinum for 20 s. Size measurements of the glass powders were performed using the specific internal tool of the software of the microscope (SmartSEM Version 6.00 with Service Pack 4).

### 2.5. Stability Test

As already demonstrated by previous works [26], the stability of the BGs in electrospinning solvents plays a pivotal role in the final properties of the composites. In order to evaluate the stability of the properties, in particular of the bioactivity, of STe0 and STe5 even after immersion in the electrospinning solution, a stability test was carried out. Briefly, both STe0 and STe5 powders were immersed for up to 1 h in AA. The acid was then removed by centrifuging and finally the glass powders were left to dry. When dried, AA-treated glass powders were characterized using FESEM-EDS and in vitro bioactivity test, as reported in [18]. Acetic acid was selected for this test, because it is a very common solvent for the synthesis of composite fibers by green electrospinning methods [27,28,29].

### 2.6. Morphological Characterization of the Composite Fibers

The selected fibrous samples were morphologically characterized using a scanning electron microscope (FESEM Auriga, Carl-Zeiss, Germany), both in Inlens and in backscattering. Samples were prepared for FESEM analysis by attaching them to a FESEM stub with double-sided carbon tape and sputtering them with a layer of gold. Based on the observation of fiber morphology and glass distribution, the best electrospinning parameters for the synthesis of composite fibers containing 5 wt%, 10 wt% and 20 wt% of STe5 were chosen. The diameter of 30 fibers of each composite fibrous mat were measured using the software ImageJ (version 1.54p, NIH, Bethesda, MD, USA) on FESEM micrographs, and then the averaged diameters were calculated and compared, in order to evaluate the effect of the BG incorporation on fiber diameter.

### 2.7. Wettability Measurements

The wettability and hydrophilicity of the electrospun fibrous membranes were evaluated by measuring the contact angle (CA). In this case, a drop of distilled water was used and both the dropping and the measurements were automatically performed using the CA device Krüss DSA30 (Hamburg, Germany). In detail, a small piece of each tested fiber was carefully cut and placed on a support as flat as possible, then 3 μL of distilled water was dropped onto the fibers and finally the angles between the fiber surfaces and the drops were calculated using the drop shape analyzer. Three measurements were performed for each type of electrospun fiber.

### 2.8. Mechanical Characterization

The mechanical properties of the electrospun fibers were evaluated by performing uniaxial tensile tests (INSTRON 5967, Instron, Boston, MA, USA) using a 100 N load cell under a cross-head speed of 10 mm/min, in air and at room temperature. In order to carry out the analysis, the fibrous membranes were cut in rectangular samples with dimensions of 3 × 10 mm^2^ and then fixed on a paper frame, as reported in [30]. Before carrying out the analysis, the thickness of each tested electrospun sample was calculated, as the average of three measurements, performed with a digital micrometer having a precision of 1 μm.

### 2.9. Antibacterial Properties Evaluation

In order to preliminarily estimate the antimicrobial properties of the obtained material, composite fibers were cut in the form of circles with a diameter of 1 cm and placed on Mueller Hinton agar plates, previously inoculated with 1.5 ×10^8^ CFU/mL of non-pathogenic strains of *Staphylococcus epidermidis* (Gram-positive, ATCC14990). Plates were then incubated in a static incubator at 35 °C for up to 24 h. To prepare the starting inoculum bacterial suspension, as reported in [31], lyophilized *S. epidermidis* were added in Mueller Hinton broth and incubated overnight at 35 °C in a static incubator. Blood agar plates were then inoculated with this bacterial broth and incubated at 35 °C for 24 h. After 24 h, few bacterial colonies were collected from these primary bacterial cultures and added to Mueller Hinton broth until a McFarland index of 0.5 (corresponding to 1.5 × 10^8^ CFU/mL) was reached. The bacterial solution was spread on Mueller Hinton agar plates; samples were placed onto the agar plates and incubated at 35 °C for 24 h. Finally, the inhibition zones around the specimens were evaluated and measured.

## 3. Results and Discussion

### 3.1. Morphological Characterization of BG Powders

**Figure 1:**
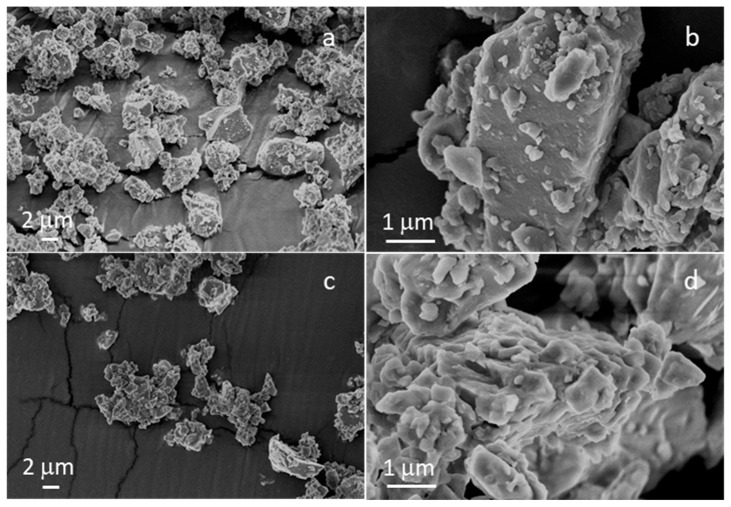
FESEM micrographs of (**a**,**b**) STe0 and (**c**,**d**) STe5.

Figure 1 shows the morphologies of the milled and sieved powders of STe0 and STe5. In agreement with previous experimental studies of these glass compositions [18], both types of glass powders were characterized by the typical sharp-cornered morphology of ball-milled glasses.

As expected, the grain size of both STe0 and STe5 powders was <20 µm, with a large portion < 5 µm. On the basis of previous results in the literature related to composite electrospun fibers containing glass powders with similar average size [28,32,33], such powder dimensions were expected to ensure a good incorporation of the glass fillers in the electrospun composites, which are the final desired material of the actual experimental study.

### 3.2. Stability Test

**Figure 2:**
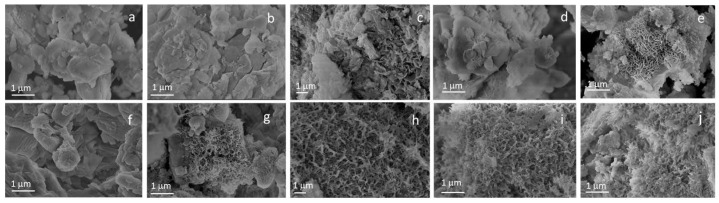
FESEM micrographs of STe0/AA SBF 0 day (**a**), 1 day (**b**), 3 days (**c**), 7 days (**d**), 14 days (**e**) and STe5/AA SBF 0 day (**f**), 1 day (**g**), 3 days (**h**), 7 days (**i**), 14 days (**j**).

The stability test showed that STe0 and STe5 maintained their good bioactivity (previously proved by Miola et al. [18]) after the immersion in AA (Figure 2). The presence of HAp was clearly evident in FESEM micrographs from the first day of immersion in SBF, demonstrating that these glasses were highly bioactive even after immersion in AA.

Initially, small HAp needles were visible (Figure 2b,c,g,h), which developed into the typical morphology of needle-like crystalline HAp grown on a bioactive glass surface, progressively increasing their size, up to 14 days of immersion in SBF (Figure 2e,j). These results confirmed the previous experimental observations of Miola et al. [18], showing that the addition of 5 mol% of TeO_2_ to the glass composition did not hinder its bioactivity. In addition, the immersion in AA (aimed to simulate the interaction between the BG fillers and the electrospinning polymeric solution, used for the synthesis of the composite fibers) did not compromise the in vitro bioactive performance of either the Te-doped BG (STe5) or its control undoped BG (STe0).

### 3.3. Morphological Characterization of Composite Fibers

**Figure 3:**
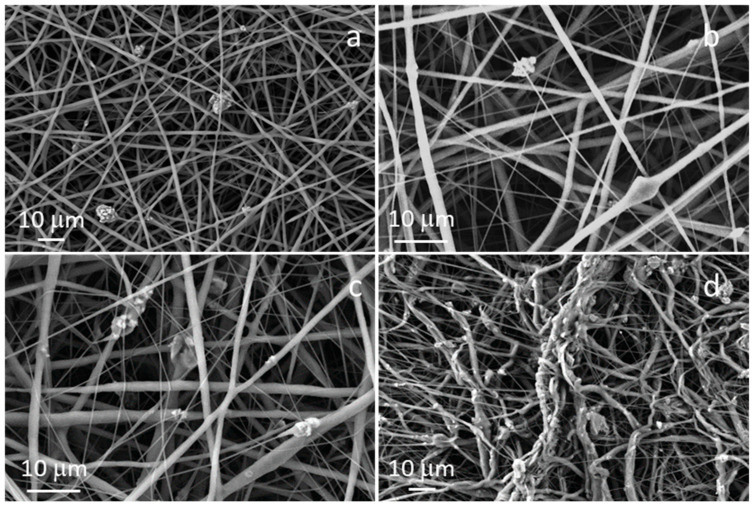
FESEM images of: (**a**) PCL/AA/STe5(5%), (**b**) PCL/AA/STe5(10%), (**c**) PCL/AA/STe5(20%) and (**d**) PCL/AA/STe5(30%).

The morphology of the composite fibers is reported in Figure 3. Composite fibers containing the lowest concentration of STe5, in detail 5 wt% (named as PCL/AA/STe5(5%)) were successfully fabricated using 15 kV as applied voltage, 11 cm as working distance and 0.4 mL/h as flow rate, maintaining the same flow rate used for pure PCL fibers (PCL/AA). In the case of composite fibers containing higher amounts of glass, in agreement with the literature results [34], it was necessary to increase the flow rate.

Composite fibers containing 20 wt% and 10 wt% of STe5 powders, electrospun using 15 kV as applied voltage, 11 cm as working distance and 1.3 mL/h as flow rate (named PCL/AA/STe5(20%) and PCL/AA/STe5(10%)) showed a better morphology and better glass distribution when compared to the fibers containing 30 wt% (named as PCL/AA/STe5(30%)), as better explained in the following paragraphs.

Regarding the glass concentration, FESEM observations showed that a concentration (wt%) of 30 was too high for the actual composite system, leading to the synthesis of fibers with a very high surface roughness (which was visible even with the naked eye, Figure 3d). This roughness was caused by both glass aggregation and the presence of aggregated fibers. The formation of glass aggregates was probably triggered by both the high amount of glass powders and the high flow rate used for electrospinning the PCL solution containing 30 wt% of STe5 (1.6 mL/h).

Good glass incorporation and size distribution of the fibers in the composite mats were obtained with a glass concentration of 5 wt%, 10 wt%, and 20 wt% (Figure 3a–c). In all these cases, using the ad hoc selected electrospinning parameters, glass powders were both incorporated into the fibers and dispersed in the polymeric matrix, on the surface of the fibers. In any case, 20 wt% seemed to be the most promising concentration in terms of glass incorporation.

These results can be better understood by observing the average values of the fiber diameters, reported in Table 1. The calculated average diameters showed a slight tendency to increase with increasing glass concentration, in agreement with previous results of Liverani et al. [23]. In addition, it could be observed that all diameter values were about 1–2 µm, confirming results from the literature obtained with the same polymeric system (PCL/AA) [25,26].

**Figure 4:**
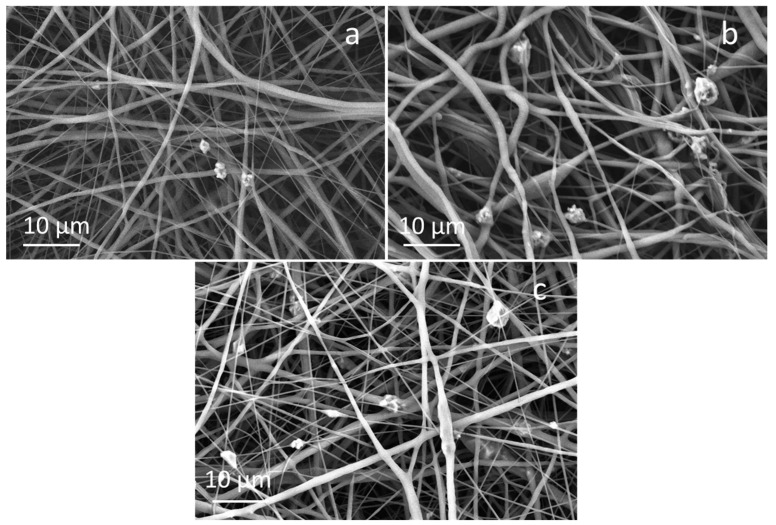
FESEM images of (**a**) PCL/AA/STe0(5%), (**b**) PCL/AA/STe0(10%) and (**c**) PCL/AA/STe5(20%).

FESEM micrographs of composite fibers containing 20 wt%, 10 wt% and 5 wt% of the control glass (STe0) are shown in Figure 4.

In agreement with results obtained for PCL/AA/STe5(20%) and PCL/AA/STe5(10%), using 15 kV as applied voltage, 11 cm as working distance, and 1.3 mL/h as flow rate, the fiber diameters seemed to be quite homogeneous. As can be observed, glass incorporation was successful but glass dispersion was not completely uniform, with some glass agglomerates. The diameter of the fibers containing the STe0 glass is slightly smaller than that observed for the fibers containing the STe5 glass (Table 2), but the differences are within the variability of the measurement method.

### 3.4. Wettability Measurements

**Figure 5:**
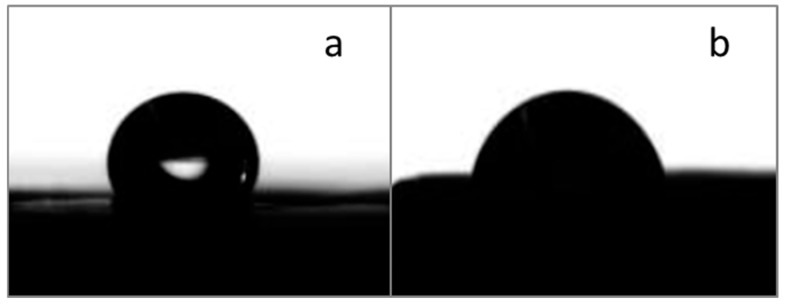
CA measurements on a pure PCL/AA fibrous mat (**a**) and a composite one, in detail, a fibrous mat of PCL/AA/STe0(5%) (**b**).

The PCL fibers showed a contact angle (CA) of 100 ± 1 (Figure 5a), in agreement with values described in the literature, which report a value between 104–140° [33,35]. The incorporation of BG powders reduced the CA, thus increasing the fibers’ wettability [36]; the obtained values were variable, but all samples displayed a CA lower than 90° and therefore hydrophilic properties (Figure 5b, PCL/AA/STe0(5%) as example). The increase in wettability is an aspect considered beneficial, as it has been demonstrated in the literature that greater hydrophilicity positively affects the adhesion and proliferation of eukaryotic cells [37].

### 3.5. Mechanical Characterization

The results of mechanical tests performed on neat PCL/AA fibers, STe5 containing fibers and PCL/AA/STe0(20%) are reported in Table 3. A reinforcing effect was noted with both STe0(20%) and STe5(20%) incorporation compared to pure PCL fibers, although the enhancement of the fibers’ mechanical properties is higher with STe5 compared to STe0 addition. When comparing the mechanical properties of composite fibers with varying amounts of STe5 powders, a decrease in mechanical performance was noted at up to 10% glass content, consistent with findings in the literature showing lower ultimate tensile strength (UTS) owing to BG’s weakening effect on polymer chain interactions [38,39]. Conversely, a reinforcing and stiffening enhancement was observed with 20% glass content, which could be explained by the higher flow rate used for the optimal fabrication of these fibrous mats, compared to PCL/AA and PCL/AA/STe5(5%) fibers. The higher flow rate resulted in a larger amount of material deposited onto the collector and, overall, in a thicker and more resistant mat. In further detail, while both compositions containing 10% and 20% glass were fabricated using a higher flow rate, ultimately differences in their mechanical performances can be explained by the effect of glass content on the electrospinning process itself, leading to a more conductive solution and stable jet in case of 20% glass content, and by the presence of an optimal glass content which is likely to positively affect the mechanical properties of the composite fibers, in this specific case amounting to 20%.

Lastly, it is important to note that there was a certain variability in the results depending on the portion of the mat analyzed and the specific electrospun mat, particularly with 20% glass content, where the dispersion of the BG powders within the fibers was observed via morphological examination to not be completely uniform, seemingly impacting the mechanical performance of PCL/AA/STe5(20%) and PCL/AA/STe0(20%), causing high variability both in elastic modulus (E) and ultimate tensile strength (UTS) values. Therefore, these results should be considered as preliminary investigations into the interaction between these novel Te-doped BG powders and a PCL matrix.

### 3.6. Antibacterial Test

**Figure 6:**
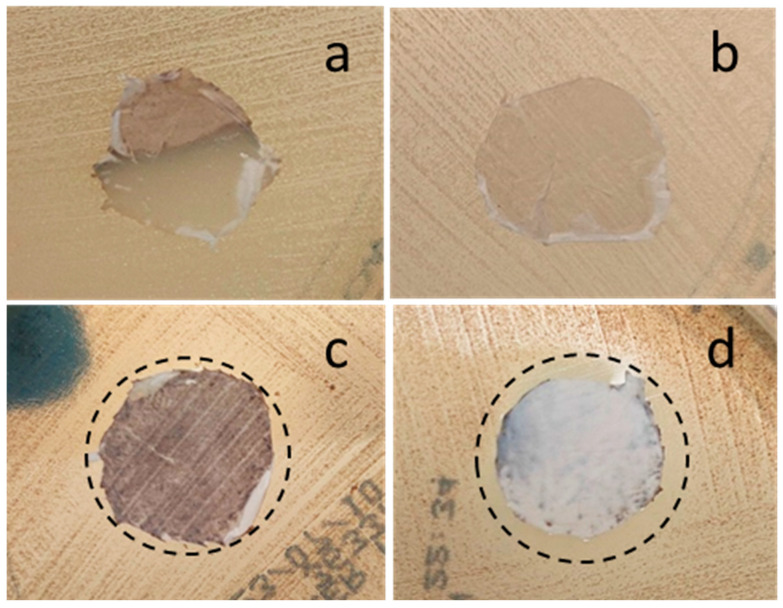
Inhibition halo test of (**a**) PCL/AA, (**b**) PCL/AA/STe5(5%), (**c**) PCL/AA/STe5(10%) and (**d**) PCL/AA/STe5(20%) fibers.

A preliminary evaluation of the antibacterial effect of Te-containing fibers was carried out by the inhibition halo test (Figure 6) using an *S. epidermidis* strain. The presence of an inhibition zone around doped composite fibers incorporating 20 wt% and 10 wt% of STe5 glass powders was seen, whereas PCL/AA/STe5(5%) did not seem to be surrounded by an inhibition zone. The size of these halos was about 4 mm and 2 mm around PCL/AA/STe5(20%) and PCL/AA/STe5(10%), respectively. As expected, the inhibition halo was absent also around the control polymeric fibrous mats, the PCL/AA. On the basis of these results, it is possible to state that the present Te-doped composite fibers possess antibacterial properties, which are given to the PCL matrix by the incorporation of antibacterial Te-doped BG powders. Indeed, the only difference between samples with and without antibacterial effects (PCL/AA/STe5(20%) and PCL/AA/STe5(10%) versus PCL/AA) was the presence of Te-doped BG powders in the composite antibacterial fibers, suggesting that the observed antibacterial action was induced by the progressive release of Te ions from the BGs and consequently from the composites. In addition, the observed dose-dependent antibacterial effect (fibers containing 20 wt% of STe5 were more antibacterial than fibers containing 10 wt% of STe5) agreed with the literature results of antibacterial tests performed on STe5 using both *S. epidermidis* and *S. aureus*, demonstrating a dose-dependent antibacterial behavior in the case of Te-containing glasses [18].

## 4. Conclusions

In this study, various amounts of bioactive glass powders containing Te were incorporated into PCL fibers through electrospinning. The research focused on the feasibility of creating innovative electrospun composites for multifunctional biomaterials with nanoscale features. The process parameters were meticulously optimized to ensure high incorporation and homogeneity. Morphological analyses confirmed the excellent spinnability of the composite fibers and the good dispersion of glass powders within the PCL fibers, achieving up to 20 wt%. The use of acetic acid as a green solvent for PCL during the electrospinning process did not compromise the bioactivity of the glasses. Preliminary mechanical tests indicated an increase in tensile strength with higher glass content, along with enhanced antibacterial properties of the composite. Further in-depth studies could be conducted on the composite material to evaluate extensively its antibacterial and antioxidant properties; however, such analyses will be the focus of future research.

## Figures and Tables

**Table 1:** Calculated average diameters of the composite fibers PCL/AA/STe5 (5–20%).

Composite Fibers	Diameter [µm]
PCL/AA/STe5(5%)	1.4 ± 0.4 μm
PCL/AA/STe5(10%)	1.3 ± 0.6 μm
PCL/AA/STe5(20%)	1.6 ± 0.9 μm

**Table 2:** Calculated average diameters of the composite fibers PCL/AA/STe0 (5–20%).

Composite Fibers	Diameter [µm]
PCL/AA/STe0(5%)	1.0 ± 0.3 μm
PCL/AA/STe0(10%)	1.2 ± 0.4 μm
PCL/AA/STe0(20%)	1.1 ± 0.4 μm

**Table 3:** Mechanical properties of PCL and composite fibers containing Te-doped BG powders.

Samples	E [MPa]	UTS [MPa]	Tensile Strain at Break [%]
PCL/AA	3.7 ± 2.0	1.0 ± 0.2	157 ± 65
PCL/AA/STe5(5%)	2 ± 0.5	0.4± 0.1	383 ± 38
PCL/AA/STe5(10%)	1.0 ± 0.4	0.2 ± 0.1	138 ± 56
PCL/AA/STe5(20%)	6.4 ± 5.2	1.8 ± 1.6	168 ± 87
PCL/AA/STe0(20%)	4.8 ± 4.6	1.2 ± 0.7	82 ± 16

## Data Availability

Data are contained within the article.
